# Building collective power in citizen-led initiatives for health accountability in Guatemala: the role of networks

**DOI:** 10.1186/s12913-020-05259-6

**Published:** 2020-05-13

**Authors:** Alison Hernández, Anna-Karin Hurtig, Isabel Goicolea, Miguel San Sebastián, Fernando Jerez, Francisco Hernández-Rodríguez, Walter Flores

**Affiliations:** 1 Center for the Study of Equity and Governance in Health Systems (CEGSS), 11 calle 0-48 Zona 10, Edificio Diamond, oficina 504, Ciudad de Guatemala, Guatemala; 2 Division of Epidemiology and Global Health, Umeå University, Umeå, Sweden; 3 Department of Computing Science, Umeå university, Umeå, Sweden

**Keywords:** Health accountability, Collective power, Citizen-led initiatives, Networks, Guatemala

## Abstract

**Background:**

Citizen-led accountability initiatives are a critical strategy for redressing the causes of health inequalities and promoting better health system governance. A growing body of evidence points to the need for putting power relations at the forefront of understanding and operationalizing citizen-led accountability, rather than technical tools and best practices. In this study, we apply a network lens to the question of how initiatives build collective power to redress health system failures affecting marginalized communities in three municipalities in Guatemala.

**Methods:**

Network mapping and interpretive discussions were used to examine relational qualities of citizen-led initiatives’ networks and explore the resources they offer for mobilizing action and influencing health accountability. Participants in the municipal-level initiatives responded to a social network analysis questionnaire focused on their ties of communication and collaboration with other initiative participants and their interactions with authorities regarding health system problems. Discussions with participants about the maps generated enriched our view of what the ties represented and their history of collective action and also provided space for planning action to strengthen their networks.

**Results:**

Our findings indicate that network qualities like cohesiveness and centralization reflected the initiative participants’ agency in adapting to their sociopolitical context, and participants’ social positions were a key resource in providing connection to a broad base of support for mobilizing collective action to document health service deficiencies and advocate for solutions. Their legitimacy as “representatives of the people” enabled them to engage with authorities from a bolstered position of power, and their iterative interactions with authorities further contributed to develop their advocacy capabilities and resulted in accountability gains.

**Conclusions:**

Our study provided evidence to counter the tendency to underestimate the assets and capabilities that marginalized citizens have for building power, and affirmed the idea that best-fit, with-the-grain approaches are well-suited for highly unequal settings characterized by weak governance. Efforts to support and understand change processes in citizen-led initiatives should include focus on adaptive network building to enable contextually-embedded approaches that leverage the collective power of the users of health services and grassroots leaders on the frontlines of accountability.

## Background

Health inequalities are manifestations of underlying power imbalances and accountability failures. Citizen-led accountability initiatives are a critical strategy for strengthening collective efforts to redress the causes of health inequalities and promote better health system governance. These kinds of initiatives may employ different tactics and approaches like citizen monitoring, community scorecards, campaigns, and advocacy to draw authorities’ attention and seek solutions to the neglected needs of disadvantaged communities [1, 2]. They contribute to redressing health inequalities by putting users of services, grassroots leaders and organizations on the frontlines of accountability, where they engage with state authorities as advocates for their own needs and rights. Ensuring accountability at the local and subnational levels of service delivery is crucial to attain the Sustainable Development Goals’ ethical imperative to “leave no one behind” [3]. This is no easy task as health inequalities affecting subpopulations are often closely linked to intersecting conditions of geographical isolation, poverty, historically entrenched norms of social exclusion, and weak state institutions [4]. Citizen-led initiatives directly engage these conditions, enabling those affected to be active agents for change in the institutions and decisions that influence their lives, rather than passive recipients of aid and interventions.

Since the early 2000s, citizen-led accountability initiatives have gained prominence, and a growing body of evidence points to the centrality of context-specific political and power relations for understanding and leveraging their influence [5–7]. In contexts of entrenched inequalities, these initiatives face an uphill battle to overcome power differentials and attempt to hold the state accountable. While the nature of the problems and local conditions they confront varies, research points to importance of iterative cycles of adaptive action in enabling citizens to build their collective power, or the power that results from building consensus and involving more people in a social process [8–11]. These cycles build both their power to act and their power to act in concert with others, which correspond to “power to” and “power with” as described by Gaventa [12]. This combination is important because empowerment, or power to, alone is not sufficient for winning rights [13]. Power with refers to “the synergy which can emerge through partnerships and collaborations with others or through processes of collective action and alliance building” [12]. Sociopolitical conditions, such as receptivity of authorities to engagement and constraints in the broader state system, also influence citizens’ potential to attain responses and solutions to their problems. The iterative cycles of engaging with these conditions and adapting their strategies can help develop the political capabilities of citizens over time and open up possibilities for expanding their agency [2].

The Center for the Study of Equity and Governance in Health Systems (CEGSS by its initials in Spanish) has been supporting the development of citizen-led initiatives to redress health system inequities in rural, marginalized communities of Guatemala since 2006. Their work is based on a rights-based model that focuses on activating the collective power of rural, excluded citizens to demand health accountability [14]. This work started in eight municipalities and has grown to include 35 municipalities in five provinces that are mostly indigenous and have high rates of poverty. Initially, CEGSS’ approach to supporting groups of citizen leaders focused on capacity-building in human rights, legal frameworks, public policy, participatory monitoring techniques, negotiation, and advocacy. As these groups have become increasingly independent in mobilizing action, their efforts to gather evidence from users of services and confront health system problems with authorities follow varying pathways in relation to sociopolitical conditions [15]. In order to continue leveraging the collective power of these grassroots citizen-led initiatives to achieve systemic and lasting impacts, CEGSS’ current approach depends on understanding and supporting adaptive, contextually-embedded processes of accountability action at the municipal, provincial and national level [11].

The case of CEGSS’ work reflects the recognized need for putting power and sociopolitical context at the forefront of understanding and operationalizing citizen-led accountability, rather than technical tools and best practices [5, 16, 17]. Researchers and practitioners emphasize the centrality of relationships and state-society interactions and the importance of adaptive learning and context-fit approaches in generating shifts in citizen power in accountability ecosystems [6, 9, 10]. Recent studies have pointed to network- and coalition-building as critical capacities that enable bottom-up accountability initiatives to connect to a broader constituency and gain influence when engaging with authorities [2, 15]. Ramalingham et al also more generally describe the nature of accountability as “an emergent result of complex interactions between multiple actors” and posit that “enlarging accountability requires attending to the web of relationships between parties and the resources they bring to the setting” [18]. Empirical studies from related fields of community development and health promotion demonstrate the potential of network mapping for visualizing actors’ linkages and positions, stimulating learning, and eliciting actionable knowledge [19–21]. However, there are few studies of citizen-led initiatives that apply a network lens to examine how these emergent webs of relationships and interconnections contribute to their capacity to influence accountability [22].

Applying a network lens to the question of how citizen-led initiatives build collective power offers the possibility to examine the invisible structures that link actors up in action to confront accountability failures. In this study, we use network mapping and interpretive discussions to examine relational qualities of citizen-led initiatives’ networks and explore the resources they offer for mobilizing action and influencing health accountability. Data was collected in three rural municipalities in Guatemala. By examining patterns in the networks’ interconnections and their interactions with authorities, we aim to deepen understanding of how grassroots citizen initiatives for accountability develop collective power to generate change in rural health systems. This study is intended to inform researchers, practitioners, donors, and other stakeholders interested in strengthening bottom-up accountability initiatives about how network qualities and resources enable contextually-embedded approaches to redress health system failures in rural, marginalized communities.

## Methods

### Study context

Health indicators and inequalities in Guatemala are among the worst in the region. Chronic malnutrition affects 50% of children under five, and the maternal mortality ratio is estimated to be 290 per 100,000 live births after adjusting for underreporting [23]. Among indigenous peoples, who make up around half of the population of 15.6 million, these rates are nearly twice as high compared to the non-indigenous population [24]. These inequities are a manifestation of social and political processes of marginalization that stem from economic exploitation, military dictatorships and a 36 year-long internal war that ended in 1996 and left 200,000 dead or disappeared, most of them indigenous [11]. Indigenous populations are concentrated in rural areas where health services are provided almost exclusively by the public health system. The disproportionate burden of morbidity and mortality they bear is further explained by the health worker deficits, frequent stock-outs of medicines and other materials, inadequate infrastructure, and the disrespectful and abusive treatment that characterize the public health services [25]. These health system failures are reflective of Guatemala’s governance environment, which is among the weakest in the region according to the World Bank’s Worldwide Governance Indicators for the period 1996–2015 [26]. Low governance scores indicate the state’s shaky capacity to carry out basic functions, like revenue collection and basic service provision, and to implement broad-base policy and public management reforms.

Social participation in governance is guided by a legal framework that established a structured scheme of development councils from the community to the national level, as well as a decentralization act that increased the power and responsibilities of municipal mayors and municipal councils [27]. These laws specify that community-level authorities, including community development council leaders and auxiliary mayors, should have a voice in municipal decision-making forums, however de facto barriers like clientelism, corruption, and transportation costs often prevent fair and effective use of this space [28]. Administrative authority is largely decentralized to regional health offices at the provincial level, where the planning, coordination and evaluation of national health programs are managed. Health service delivery via a central health center, or in some cases a district hospital, and peripheral health posts is overseen by a district manager at the municipal level [29]. Municipal governments are also responsible for coordinating with district health authorities and allocating a portion of their budget to health service needs such as refurbishing of healthcare facilities, ambulance and support personnel (drivers, auxiliary nurses) if needed.

Across the 35 municipalities where CEGSS supports accountability initiatives that operate in these conditions, there are over 120 citizen leaders mobilized in the role of community defender for the right to health (from now on referred to as defenders). These defenders are volunteers nominated by their communities and many also have roles as community-level authorities and/or in grassroots organizations. They receive on-going training and support to develop and implement knowledge and skills to mobilize accountability action in three main domains – grassroots network development, monitoring of health services, and engaging with authorities [15]. Most of the municipalities have three or four active defenders who organize monitoring visits to health facilities, collect user reports of problems, and advocate for solutions with authorities at different levels, including mayors, municipal councils, health district managers, regional health directors, Ombudsmen, public prosecutors, and provincial and national officials. The groups of defenders implement these actions in coordination with the support of local collaborators, who typically have roles as community authorities or with other groups and organizations involved in collective action. It is these groups of defenders and their collaborators that we refer to as municipal-level citizen-led initiatives. In recent years, CEGSS’ support has also focused on linking up these groups in a national network (REDC-SALUD as per its name in Spanish) that serves as a platform for analyzing the root causes of their common struggles and planning strategic action to influence systemic change, however, this national network is not in focus in this study [11].

### Study design

A network view is useful for focusing attention on the invisible structures and relations that connect citizen-led initiatives to their social environment, and for gaining insight into the resources that initiatives offer for mobilizing collective action. The relational and structural qualities of the initiative’s internal and external interactions reflect how they function to unite individuals, organizations, and communities in collective efforts to make their problems visible and seek solutions [30]. These internal interconnections also reflect their access to two kinds of resources for building power: the collective social capital represented by the nature of their ties and interactions, and the linkages that initiative participants provide to different sectors [31]. An initiative’s interactions with authorities over time are also relevant for understanding how their potential to influence accountability is shaped by their sustained engagement with the power dynamics of the local sociopolitical landscape [16].

In this study, we combined network mapping and interpretive discussions in a mixed methods case study design to gain a more holistic view of municipal-level citizen-led initiatives and their collective action. Case study design permits combining different information sources and data collection methods to answer how and why research questions that deal with complex embedded phenomena that are difficult to separate from their context [32]. An adapted social network analysis questionnaire was used to generate network maps that provided a visualization of who the defenders were collaborating with and how they were linked up in collective action. Our analysis of these maps was enriched by the interpretive discussions, which provided insight into initiative participants’ views on what their interactions represented. The combination of these data collection methods enabled us to make network qualities of the initiatives visible and incorporate participant perspectives in our interpretation of their significance.

We selected cases of municipal-level citizen-led initiatives where the defenders had a strong network of community involvement in their action for accountability. Organizational monitoring data describing the defenders’ activities and interactions with authorities from 2013 to 2015 provided insight into the level of involvement of other community leaders across all of the municipalities where CEGSS was active [15]. A set of cases with strong community involvement in this time period was identified and the list was presented to CEGSS field staff whose role is to support and monitor the defenders’ planning and action. Through discussion with the field staff, some potential cases where defenders’ work had declined for different reasons were eliminated and others were added where activities and community involvement had increased. These discussions led to the selection of three cases - Concepcion, Santana and Tolima (pseudonyms have been used to protect confidentiality) - each coming from different regions of the country where initiatives are active. The receptivity of the defenders to learn about their network and participate in interpretation of the results was also an important selection criterion. An invitation letter was sent explaining the study and what their participation would entail, and the three selected defender groups agreed to participate.

The study proceeded in four steps: network data collection, generating maps, interpretive sessions, and case analysis. These steps are described below.

### Network data collection

In order to collect network data, the defenders were asked to invite their collaborators to participate in a workshop about network strengthening in early 2017. Collaborators were defined as the people who support them in their work for health accountability, and the defenders were responsible for recruitment. While one or two of the collaborators they invited were not able to participate, the groups of participants provided a snapshot of the segments of civil society that the initiatives mobilize. During the workshop, CEGSS field staff led discussions about the importance of networks and strategies for strengthening them. The research team then explained the study procedure, how the information would be handled, and obtained informed consent. The sessions were conducted in Spanish with translation to the local indigenous language facilitated by the CEGSS field staff when needed.

A social network analysis questionnaire developed with Open Data Kit (ODK) was administered on tablets [Additional file 1 – Social network analysis questionnaire]. First the names of participants were entered in the questionnaire, and they were each asked about their own affiliations and their relational ties with each of the other participants. The questions developed for this study focused on type of relationship, frequency of communication, and history of collaboration, and options for answers were provided on a scale. Then we gathered the names of the authorities with whom they had interacted about health system problems in the past year and asked each participant questions about the frequency and nature of their interactions. In order to avoid the risk of having unintended influence on the defenders’ processes with authorities, particularly those who were less receptive, we did not seek authorities’ responses to these questions. So the view we obtained of these interactions was based only on the accounts of initiative participants. Field notes from the workshops provided additional information about who the participants were and the content of the group discussions.

### Generating maps

The network data was exported from the ODK platform and converted to matrices of participants’ responses for each question. In the case of the questions that participants answered about each other, the matrices were one-mode, meaning that the rows and columns of names were the same. The questions about interactions with authorities were two-mode matrices, with participant names as rows and authority names as columns. In the case of the one-mode matrices, we checked for symmetry (match) between the participants’ responses about each other. In the majority of comparisons, there was a match in the relation reported by the dyad of participants. In the case of discrepancies, we followed the union rule and assigned a single value to reflect the strength of the tie based on the higher score [33].

SocNetV software was used to generate maps and analyze the ties among participants [34]. The density and network centralization were calculated. Density indicates the interconnectedness of the network, with the number of existing ties expressed as a proportion of all possible ties. High density indicates that many participants have interactions, while low density reflects that fewer participants interact with or know each other. Centralization refers to the extent to which the network is dominated by a single or a few actors. In a highly centralized network, one or a few actors would serve as the main point(s) of connection for the group, while in a decentralized network, the ties connecting the group are more evenly distributed among participants. Group degree centralization scores were calculated with values ranging from zero, where all nodes have equal ties, to one, where one node dominates the others. Maps depicting participant ties with authorities were also generated and preliminary analysis of these maps focused on the distribution of ties with authorities among participants. In-depth familiarization with participant profiles and their roles (e.g. organizations they belong to, positions in the community) was also critical in our initial interpretations of the network maps in preparation for the next step of the study.

### Interpretive sessions

The interpretive sessions took place around 6 months later in mid-2017, and the participants were invited to discuss the results from the previous session and develop an action plan for strengthening their network. Overall there was a 25% decline in the number of participants in this step. The defenders reported that some of those who did not join the session had a schedule conflict with other civil society or municipal meetings, while a few had become less active in their collaborator role. The procedure for the session and the importance of confidentiality were explained, and informed consent was recorded. Participants reviewed the maps of their network and a member of the research team guided discussion about what the ties represent, the focus and motivation of their history of collaboration, how they communicate to coordinate action, and the focus and nature of their interactions with authorities. The sessions ended with reflection on the insights gained about their networks and formulation of an action plan to work together to strengthen them. Follow-up interviews were also conducted with the lead defenders and the field staff who worked most closely with each municipality to gather further information about the topics covered in the group discussion. The discussions and interviews were recorded and transcribed.

### Case analysis

The first step in case analysis was to order and synthesize the qualitative data using thematic analysis [35]. This process began with familiarization with the data and development of a set of 12 thematic codes, such as history of collaboration and influence with authorities. These codes were applied to the group discussions, interviews, and field notes using NVivo 11 qualitative data analysis software. Texts marked with each code were reviewed to identify cross-case patterns and refine an overarching framework of themes to guide development of the case reports.

Case reports consisted in profiles of the participant groups, maps of collaboration ties within the initiative and interactions with authorities, analysis of reported relational ties, and syntheses of the qualitative findings on motivation to collaborate, coordination of collective action, history of engagement with authorities, and plans for network strengthening. In cross-case analysis, we sought to identify patterns in the participant profile information, their relational ties, the descriptions of how they collaborate, and their respective sociopolitical contexts. Review of relational and structural qualities and accounts of their action provided insight into how these network qualities interacted with other local conditions to give rise to the initiatives’ collective action and capacity to influence accountability.

### Ethical considerations

Ethical approval of this study was granted by the Ethics Committee of the Social Science Faculty at the Universidad del Valle in Guatemala.

## Results

This section presents our findings on the network qualities of the citizen-led initiatives and their relation to mobilization of collective action and accountability outcomes in Concepcion, Santana and Tolima. First, we present a comparative overview of the network characteristics of these groups, drawing on participants’ accounts of what the ties connecting them represent. Then we explore the motivations and the issues that unite them in collaborative efforts, and we examine how the initiatives’ networks provide resources in their collective action gathering evidence of health system problems and engaging with authorities. Finally, we consider how the resources that participants gained from their networks contributed to accountability outcomes and their plans for strengthening them.

### Profile of social position of initiative participants

The groups of initiative participants gathered for the study in the three municipalities were similar in number (10–13 participants), but the profiles of the roles they held in their communities varied (Table 1). Concepcion participants included representatives from several women’s community-based organizations (CBOs), as well as actors with roles in different municipal institutions and the district nurse from the health center. In Tolima, participants from urban civil society, who were active in community organization roles, joined with indigenous authorities and members of a traditional birth attendant (TBA) organization, who were from remote villages. While in Santana, most of the participants were leaders from different small villages with supporters from urban civil society and two rural community organizations.

**Table 1:** Profile of municipalities and participants. CBO = Community-based organization, TBA = Traditional birth attendant

	Municipality profile	Role in initiative		Sex		Role in community	
Concepcion	Population: 7000 in 12km^2^	Defenders	3	F	2	Women’s CBO	2
	Poverty: 81%			M	1	Agricultural cooperative	1
		Collaborators	8	F	2	Municipal institutions	3
				M	6	Village leader	2
						Women’s CBO	2
		**Total**	**11**			District Nurse	1
Santana	Population: 8000 in 68km^2^	Defenders	5	M	5	Village leader	4
	Poverty: 85%					Urban civil society	1
		Collaborators	5	F	1	Village leader	2
				M	4	Agricultural cooperative	1
						Women’s CBO	1
		**Total**	**10**			Urban civil society	1
Tolima	Population: 27,027 in 196 km^2^	Defenders	3	F	1	Urban civil society	3
	Poverty: 96%			M	2		
		Collaborators	10	F	3	TBA association	4
				M	7	Indigenous authority	2
						Village leader	2
		**Total**	**13**			Urban civil society	2

Participants’ diverse roles provided the initiatives with connections to different segments of the population of users of services as well as authorities. The social positions of village leaders, leaders of women’s CBOs, and TBAs were useful for convening the population to raise awareness about health rights and providing a point of contact for users of services to voice a complaint. In Concepcion, the lead defender described that their collaboration with diverse actors allowed her to be a voice for health in different activities and spaces, so that their work can “connect to more people and help more people.” The TBA association in Tolima played a key role in connecting the defenders from urban civil society with the fairly large and disperse population of users of health services through their role as traditional health providers and recognized figures in the villages. The collaborators from municipal institutions in Concepcion and the indigenous authorities in Tolima were also regular participants in municipal decision-making spaces and served as a resource for gaining audience and efforts to influence local authorities. In Santana, many participants held similar roles as village leaders, but they were from four different remote communities, each with a local health post. They described that they used their space in community-wide assemblies and meetings with other community authorities to educate about rights and how to report complaints about services.

### Nature of interconnections: relational and structural qualities of initiative groups

The nature of interconnections within the groups was reflected by the density of ties, the types of relationships and frequency of communication (Table 2). In Concepcion and Tolima, the higher density scores indicated that the majority of participants knew many of the others, while in Santana there were more participants who knew few of the others. Overall, the participants were connected by a mix of strong and weak ties. Participants were more likely to describe their relation to others as distant or work-related than friendship or family. Reports of the frequency of communication between participants indicate that at least one-third of relations reported were fairly distant, with contact only one to four times in the last year. However, in Concepcion and Tolima, there was also a substantial proportion of relations (50 and 40%, respectively) where communication occurred at least monthly. These relational qualities of higher density of ties and more frequent communication indicate a greater degree of cohesiveness and a stronger history of coordination among participants in these initiatives. In Santana, the lower density of relational ties and less frequent communication reflect that participants share less collective history as a group, at least in part due to the distances between villages.

**Table 2:** Structural and relational qualities of initiative groups

	# Actors	Structure of ties		Type of relationship^a^		Frequency of communication in past year^b^	
	(# of ties)						
Concepcion	11	Density	0.84	Family	4%	1–4 times	33%
	(46)	GDC	0.20	Friend	9%	5–10 times	17%
				Colleague	27%	Monthly	35%
				Acquaintance	60%	Weekly	15%
Santana	10	Density	0.62	Family	11%	1–4 times	36%
	(28)	GDC	0.47	Friend	35%	5–10 times	39%
				Colleague	39%	Monthly	18%
				Acquaintance	15%	Weekly	7%
Tolima	13	Density	0.71	Family	2%	1–4 times	47%
	(55)	GDC	0.35	Friend	16%	5–10 times	13%
				Colleague	40%	Monthly	16%
				Acquaintance	42%	Weekly	24%

^a^In the case of relationships that fit more than one category, participants were asked to pick the type that they considered most accurate with a friend being a closer, more personal relationship than a colleague

^b^Communication was defined to include face-to-face as well as phone calls and text messaging

**Fig. 1:**
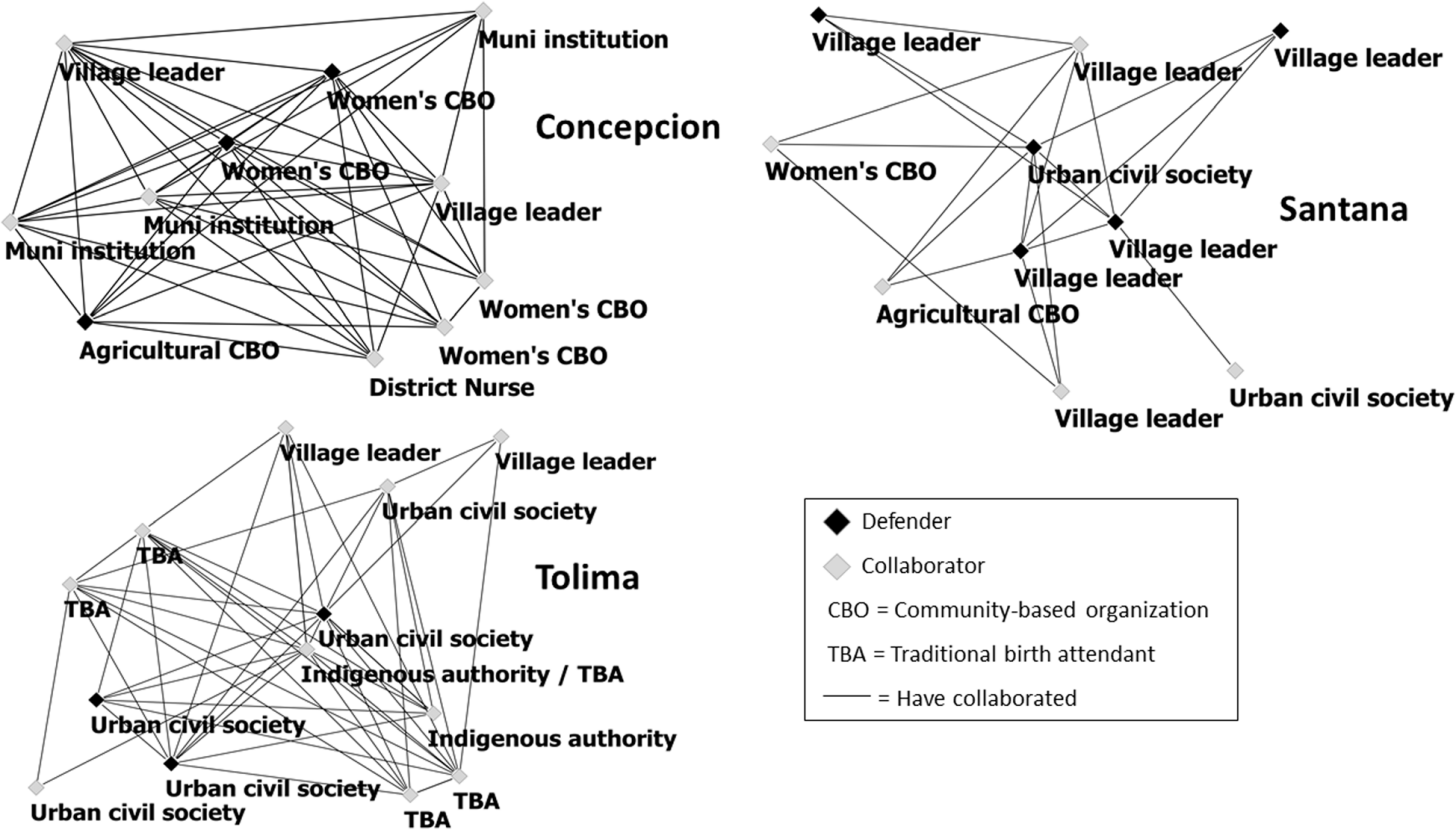
Network maps of collaboration ties. Ties between participants represent that they reported having collaborated together in efforts for the good of the community. The position of the participants in the maps reflects their centrality in the group, with those towards the center having more collaboration ties with others

The group degree centralization (GDC) scores are relevant for understanding the structure of interconnection in each group. The groups in Concepcion and Tolima had lower centralization, which reflects a more even distribution of ties among participants. Santana’s higher centralization reflected that a few participants dominated the ties more than others. The structure of participants’ ties in collaborative action are shown the network maps in Fig. 1. These maps show a higher density of ties among participants in Concepcion and Tolima, reflecting more history of collaboration within and across subgroups with similar profiles. This pattern reflected more frequent interaction in broader efforts for health among groups like the leaders from women’s CBOs with actors from municipal institutions in Concepcion and between the TBA association with urban civil society in Tolima. In Santana, the history of collaboration among participants was not as strong, with not even all defenders reporting collaboration ties with each other. The lower density and higher centralization in Santana reflect how three more central defenders played a key role in bringing the others together, who did not interact otherwise.

### Focus of collaborative efforts

Participants’ accounts indicated the histories and motivations behind the ties of collaboration shown in Fig. 1. In both Concepcion and Tolima, participants were collaborating for the right to health, but they were also collaborating in other ways. Collaboration in Concepcion was driven by a desire to see their municipality develop, and they worked together in different sectors to “tackle any problem that presents – it doesn’t matter if it is education, security, health, anything.” While in Tolima, many of the participants had roles in health promotion and they were motivated by first-hand contact with their people’s health needs and their grievances with the health system. One of the TBAs described that when you see the severity of people’s needs, “there is something in your heart that makes you stand up”. They feel an imperative to do something because “we can’t sit by and do nothing.” Their work together involved coordinating care for patients and supporting them to seek health services. The history of collaboration in Santana was more limited because the ties represented a coordination among village leaders who had not previously worked together. In this case, the group was brought together by their interest in improving the health services in their villages. They described themselves as watching out for their rights because if they do not, no one will. One defender described that it has been challenging to get more people to support their efforts due to poverty and the burden of losing a day’s work. But he states that:

*“knowing your rights is also part of (getting ahead in) life, because just look at how things are going … people are realizing about the manipulation and the corruption … Our history obligates us to fight for the rights we have as Guatemalans.”*

### Mobilization of network resources in action for accountability

Collective action for the right to health across the three municipalities had a common focus on gathering evidence of health system problems and engaging with authorities to seek resolution. The mobilization processes emerged from the interaction between the resources offered by the initiatives’ network, the dispersion of the population and health services, and the receptivity of local authorities.

Initiative participants’ connections to the population bolstered action to gather evidence of health system problems. In addition to Concepcion participants’ positions as leaders of women’s CBOs and village leaders, the small geographical area and the receptivity of district health authorities facilitated contact with users of services through regular monitoring visits at the health center. Occasionally actors from municipal institutions and village leaders accompanied the defenders on these visits to give more visibility to problems like inadequate infrastructure and lack of medicines. In Santana, even though relational ties were weaker and more centralized, the geographical distribution of the network permitted coordination of extensive documentation of problems in the health services via monitoring visits and collection of user reports. Participants in Tolima also facilitated connection to a more disperse population, with village TBAs referring the defenders to interview service users with complaints. The ties connecting the members of the TBA association and the defenders enabled the collection of many reports of abuse and mistreatment in the district hospital in Tolima. Through their role accompanying their patients, the TBAs had experienced this problem first-hand. One described that they were:

“*treated like worthless garbage … They throw people on the beds, even nude … Particularly the student doctors treat people like dogs, telling them there is nothing wrong with them. They don’t care where someone has pain*”.

The TBA collaborators provided a key connection to patients because the hospital authorities in Tolima were not open to monitoring visits.

**Fig. 2:**
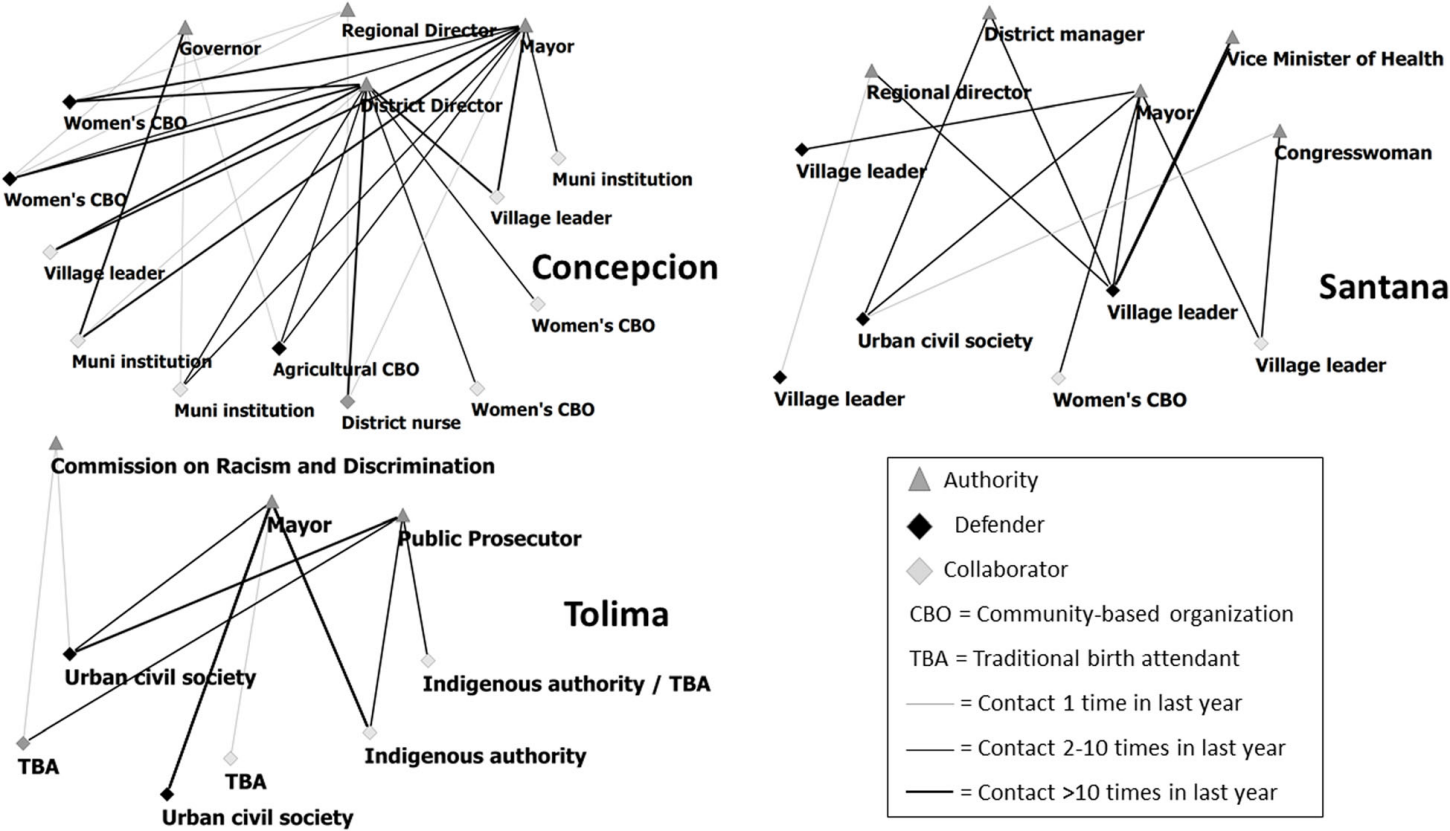
Maps of communication ties between participants and authorities. The thickness of the ties reflects participants’ reports of the number of times they had communicated with the authority in relation to advocacy in the last year

The evidence of local problems gathered through their connections with communities provided the basis for going to authorities to advocate for solutions. The maps in Fig. 2 show the variation in the participants’ engagement with authorities across the three cases. In both Concepcion and Santana, participants had interacted with municipal and district health authorities, as well as regional level authorities. Their advocacy with municipal and regional authorities focused on similar problems of medicine shortages and deficient infrastructure, and both groups had also engaged with district directors to seek response to user complaints of mistreatment. In Concepcion, defenders participated regularly in the municipal council and presented petitions for improvements to the health center and supplementing the medicine supply with backing from their collaborators. They attributed their ability to be heard in these spaces to the municipal authorities’ recognition of their role as “representatives of the people for health” and the influence of key collaborators who they mobilized to accompany them. Even though the municipal authorities were receptive to their participation, “many times they don’t fulfill their obligations”. Through the collective action of the participants, “they feel a little pressured to help the health services”. In Santana, a smaller group was engaging with authorities and their interactions were less frequent compared to Concepcion. The defenders had a position to participate in the municipal council meetings, but they were not always included and local political rivalries made it challenging to align supporters. They presented petitions for repairing the roofs of the health posts with municipal authorities as well as regional authorities. And the lead defender maintained regular communication with the regional and district health directors and vice minister of health about the status of the medicine supply in the health posts.

Interactions with authorities in Tolima took shape differently, with defenders, indigenous authorities and members of the TBA association interacting with the mayor and filing complaints in legal institutions to seek solution for the mistreatment both the TBAs and their patients were experiencing in the hospital. Even though defenders participated in municipal council meetings with some network allies, the authorities were not responsive in seeking a solution. And hospital authorities were not open to giving them audience. As an alternate route, they would support service users who were interested to file a legal complaint with the public prosecutor. The defenders and the TBA association also filed a collective grievance about the abuse and mistreatment at the hospital at the regional office of the Commission against Discrimination and Racism (CODISRA). At the time of the second study visit, a representative from CODISRA had been sent to investigate but no action had been taken.

### Value of network resources for accountability outcomes

The processes that the initiatives adopted to mobilize their networks in collective action involved long term engagement and continual adaptation of strategies. In both Concepcion and Santana, the initiatives were able to leverage community-generated evidence and iterative interactions with different authorities to gain influence. Municipal resources had been mobilized in these cases for resolving different health system problems, including infrastructure improvements and medicine supply, and the initiatives had facilitated improved coordination between municipal and district health authorities.

Concepcion’s dense mix of strong and weak ties with collaborators in positions to communicate to the population and to lend their influence to petitions with authorities enabled the initiative’s work to gain recognition and credibility in this small community. Their action plan for strengthening their network focused on coordinating intersectoral action for health with current and new collaborators, which reflected their strategies of approaching health in an integral way by “joining forces and having unity among those who work in different areas” and presenting broad backing for health issues to increase the “legitimacy and validity of their proposals”.

Santana’s network of mostly weak ties enabled the initiative to link up participants in positions to give voice to rural service users’ problems with a central subgroup that was actively communicating with authorities at different levels to coordinate support. As a group of mostly remote village leaders, they faced a greater power differential in interactions with authorities, but they contributed to an improved coordination between regional and district health authorities in medicine delivery. They attributed the influence they have gained to their way of engaging:

*“They listen because we are a group that is trained to be able to present, to listen and negotiate for what we want, and how to collaborate more than anything”.*

The extensive evidence they collected also gave them credibility as a voice of the people. Their action plan focused mainly on strengthening their community base through involving influential community leaders and developing an alliance with a rural women’s network.

In Tolima, the initiative faced more challenging local sociopolitical conditions. Though they had gathered substantial evidence and had the support of indigenous authorities and the TBA association, they had not been able to obtain a response from local authorities. The nature of their problems in the hospital reflected dynamics of racism and discrimination. They were not able to engage directly with hospital authorities, but they did seek legal recourse for several cases of rights violations and gathered reports about many more. Their action plan focused on convening influential community leaders to discuss the problem and nominate a committee to follow up on their collective grievance filed with the Commission against Discrimination and Racism (CODISRA) and seeking alliance with a regional TBA network to provide a broader base of support. Their efforts resulted in CODISRA sending another representative from the national level and an intervention with the hospital director. However, while defenders reported some improvements, the problems of mistreatment were not yet not fully resolved at the time of completion of this study.

Across the three cases, participants and CEGSS field staff reported that the maps were useful for seeing the range of actors they were working with and being able to “evaluate their relationships”. There was concern among field staff that some of the participants did not fully understand the maps, but they also expressed that the maps provided useful tools for planning action and working with the defenders to see “how their networks are doing … so they can be strengthened and their actions can have better results”.

## Discussion

This study indicated that networks play a critical role in enabling citizen-led initiatives to achieve health accountability gains in rural Guatemala. By applying a network lens to three cases of municipal-level initiatives, we identified key resources that their networks offered for shifting power in accountability ecosystems characterized by weak governance and social exclusion.

### Adaptability to context

Comparison and interpretation of the relational and structural qualities of the initiatives’ networks indicated how their collective work functioned to mobilize resources and confront power as they presented in their own social context. Variations in the density of their ties and the cohesiveness of their networks supported the notion that “with the grain” best-fit approaches are critical to enable initiatives to adapt to the realities of their environment and have a bearing on power [7, 36]. Concepcion’s and Tolima’s networks both exhibited more cohesiveness than Santana, reflecting the joined forces of women’s CBOs and municipal institutional actors and urban civil society and a TBA association working together for health. Although stronger cohesiveness is typically associated with greater solidarity and strength for collective action, Santana’s more centralized network with fewer and weaker ties illustrated that networks can provide resources in adaptive ways [37]. Weak ties enabled them to coordinate health service monitoring across remote villages, providing a different example of the recognized “strength of weak ties” that enable linkage to external resources [33]. The structural variations in the ties making up these three initiative networks reflected the defenders’ agency in bringing together actors with shared interest in improving health and health services who were linked to different resources for collective action.

### Broad base of support

Interpretation of the communication and collaboration ties represented in the maps indicated that the initiatives had been successful in linking up with a broad constituency and working collectively for common goals. The social positions of the initiative participants were key in facilitating this, as their roles as village leaders and in other organizations connected them to different population segments. These positions were useful for convening the population for awareness raising and providing points of contact with the users of rural services who experience health system failures. The legitimacy provided by this connection to the people and their collective action to document their problems was an important resource for enabling the initiatives to enter interactions with authorities from a bolstered position of power as “representatives of the people”. In Concepcion, we found that participants with roles in municipal institutions helped lend weight to their interactions and overall the participants were more active in advocacy efforts, reflecting that their network offered more resources for gaining influence and also that the local sociopolitical environment was more supportive of citizen participation. In the cases of Santana and Tolima, it was not clear that the initiative participants with linkages to decision-making spaces were an asset in helping them gain influence with authorities, and their action plans to strengthen their network included seeking alliances with influential community leaders. The context-specific evidence generated by this study was useful for informing initiative strategies to build horizontal associations with other well-positioned civic leaders as a way to broaden their support and enhance their clout with local and regional authorities. Explicit attention to how these kinds of network resources are developed is also critical for supporting CEGSS’ vertical integration strategies that link national level action with grassroots action and seek to strengthen their position with national authorities through a broad base of citizen-generated evidence and support [38, 39].

### Capabilities for influencing accountability

Beyond the numbers of people linked to the initiatives’ accountability action, the ties among participants and with authorities reflected the resource of their cumulative experience of working together to resolve community problems. These network ties provided insight into the history of collective action through which these rural citizens’ capabilities to influence accountability had developed. In Tolima, we saw how collaborative ties between urban-based defenders and the TBA association enabled the initiative to reach users of services, document, and expose abusive and discriminatory treatment, effectively overcoming the hospital authorities’ efforts to protect their personnel by not allowing monitoring. Their strategic collaboration led to an intervention by a national representative of the Commission against Discrimination and Racism. While in Concepcion and Santana, the initiatives’ evidence, alliances, and iterative interactions with authorities led to allocation of municipal resources to shore up deficits and improved coordination with regional health authorities. The initiatives’ accountability gains can be seen as relatively modest, particularly when taking into account the systemic roots of problems like drug shortages, neglected rural infrastructure, and impunity. However, it should also be noted that while outcomes like national health policy and public management reforms appear to offer further reaching impact, in contexts of difficult governance, they often face many barriers to effective implementation. In such situations, citizen-led accountability action can help create islands of more effective governance at the frontlines of service delivery and also provide a complementary path to generating the conditions that could eventually facilitate better policy implementation [36]. Additionally, “small wins” contribute to building initiative participants’ political capabilities and confidence, and they reflect incremental shifts in power in the accountability ecosystem, which are critical to sustain and leverage the impact of citizen-led initiatives as their focus expands to the national level [2, 6, 40].

### Methodological considerations

The networks of ties connecting citizen-led initiatives are “real entities whose properties are different from those of its constituent elements” [33]. In this study, our adapted social network analysis approach was valuable for shedding light on the social structures through which the initiatives’ collective action takes shape. The visual representations of networks provided a view of how participants were related through ties of communication and collaboration, and the nature of their engagement with authorities. This view was helpful from a practitioner perspective for yielding actionable knowledge, which is a virtue of network mapping that has been noted in other studies [21, 41]. However, we also acknowledge that the network maps we studied were a snapshot of the initiative participants who were able to attend the workshops in the first phase of the study, and not a full picture of everyone who was active in the initiative. This limitation reflected the necessity of presential data collection in these settings where the participants lived in remote villages and did not utilize email, which is a tool used in many network studies to facilitate fuller representation.

Discussions about the maps with participants enriched our understanding of how their interconnections facilitated building collective power, and the inductive research process also served to engage participants in reflection about their networks’ composition and how to strengthen them. It was noted that it was difficult for some participants to fully understand the maps, which reflected the challenges of making complex information accessible for populations with low literacy levels. But the overall quality of the participants’ input was sufficient to enable a more robust and holistic analysis of the function and meaning of their ties than could be gained by looking at the network data alone. In such discussions, it is also possible that power dynamics among participants may lead to some interpretations coming through more strongly than others. In this study, the defenders and collaborators with more central positions in the network also tended to be more vocal in the discussions, reflecting their leadership in the initiative and in the community. The views of those orchestrating collective action were of key interest for our study, and we may have less complete information about the perspectives of participants in a more supporting role. Consideration of the unique views and knowledge that more peripheral (less connected) network members may hold can be useful for designing an interpretive discussion guide that encourages their participation.

Our approach of combining network mapping and interpretive discussions holds potential for supporting adaptive learning processes and deepening understanding of how network structures provide the conditions from which collaborative, bottom-up initiatives for social change grow in other settings. Adaptations of this approach may include more in-depth analysis of network qualities, such as the role of participants with bridging and brokerage positions [19]. Longitudinal network studies can also be useful for moving beyond the snapshot view to examine the stability of the ties over time and the evolution of the webs of relationships that connect citizens and the state in efforts to enhance accountability in rural health services and other sectors [42]. Attention should also be given to adapting and breaking down the visual representations in the maps to make them more accessible for populations with limited formal education.

The interpretive discussions and the action plans developed by initiative participants themselves allowed CEGSS to provide context-specific support for network strengthening. This study informed organizational learning by shedding light on the fact that although the three municipalities have very similar social, economic and ethnic indicators, their network strategies and needs for support are very different. As a result of this study, CEGSS is implementing procedures to understand citizen-led initiatives’ networks in other municipalities and at the national level in order to tailor the technical assistance and support that they provide.

## Conclusions

When citizen-led initiatives act to redress the medicine stock-outs, deficient infrastructure and abusive treatment they experience in rural health services, they confront the embedded sociopolitical forces that perpetuate health inequalities. In this study, we used network mapping and interpretive analysis to take stock of the resources that the initiatives’ networks offered for building collective power and influencing accountability. Our findings provided evidence to counter the tendency to underestimate the assets and capabilities that marginalized citizens have for building power, and reaffirmed the idea that best-fit, with- the-grain approaches are well-suited for highly unequal settings characterized by weak governance.

We gained key insights into the role of relational and structural network qualities in citizen-led initiatives’ efforts to gain influence and generate change in the rural health system. Firstly, our findings showed that more is not necessarily better when it comes to density of ties or cohesiveness. Rather, variations in these three initiatives’ relational qualities reflected the different resources and capabilities their networks offered and their agency in weaving them together in response to local circumstances. Secondly, the connections their networks facilitated to a strong community base of support was fundamental to their ability to mobilize collective action to give voice and demand attention to rural health system failures. This grassroots support helped the citizen-led initiatives gain recognition and legitimacy as representatives of the people. Finally, while the three initiative cases under study managed to achieve modest improvements in local health services, our study pointed to the importance of focus on enhanced political capabilities and strategic alliances alongside health service improvements as indicators of power shifts in the accountability ecosystem.

Given the scale of the entrenched inequalities that citizen-led initiatives seek to disrupt, incremental and sustainable gains in “power with” are critical to enable grassroots leaders to engage with authorities from a strengthened position. To advance on such gains, local initiatives must link up and leverage their resources by building alliances with civil society organizations and progressive authorities at the national level. A year after this study was completed, municipal-level initiatives secured and are currently implementing a letter of agreement with the National Ombudsman office and established collaboration with national level think tanks. This example of scaling-up of citizen-led accountability through adaptive network building appears to be contributing to long-term processes of social change by uniting a broad base of support and enhancing communities’ capabilities to influence authorities, and it may be of relevance to other countries that face a similar reality and challenges to those in Guatemala.

## Supplementary information

**Additional file 1.** Social network analysis questionnaire.

## Data Availability

Network and qualitative datasets generated and analyzed in this study are available from the corresponding author on reasonable request.

## Abbreviations

CODISRA: Comisión Presidencial contra la discriminación y el racismo (Presidential Commission against Discrimination and Racism)
CBO: Community based organization
CEGSS: Centro de Estudios para la Equidad y Gobernanza en los Sistemas de Salud (Center for the Study of Equity and Governance in Health Systems)
GDC: Group degree centralization
ODK: Open Data Kit
TBA: Traditional birth attendant
