# Supplementary material for: Building collective power in citizen-led initiatives for health accountability in Guatemala: the role of networks
Source: BMC Health Serv Res. 2020 May 13;20:416. doi: 10.1186/s12913-020-05259-6 (PMC7218564; doi:10.1186/s12913-020-05259-6)
Supplement: Supplementary file 1 — Additional file 1. Social network analysis questionnaire. [file 12913_2020_5259_MOESM1_ESM.docx]

**Additional file 1: Social Network Analysis Questionnaire**

**Part 1: Relations among collaborators**

*I am going to ask you some questions about your relationship to each of the other defenders and collaborators who are gathered here. I will tell you the name of the person and then ask you the questions about that person. Then we will go on to the next person and answer the same questions about him/her, and so on, until we complete the list.*

1. Which best describes your relationship with (this person)?

a. Family b. Friendship c. Colleague d. Acquaintance e. None

*If None, skip questions 2-6 and go on to the next person.*

2. In the past year, about how many times have you communicated face-to-face or by phone with (this person)?

a. 1x / week b. 1x or more / month c. 5 - 10 times d. 2 - 4 times

e. 1 time f. Never

3. Have you ever collaborated with (this person) in efforts to improve situations that are of benefit to the entire community or population?

a. Many times b. A few times c. A little d. None

4. In the last year, approximately how many times have you collaborated with this person in actions to promote training and education on the right to health at the community level

a. 1x / week b. 1x or more / month c. 5 - 10 times d. 2 - 4 times

e. 1 time f. Never

5. In the last year, approximately how many times have you collaborated with this person in actions to collect evidence and complaints

a. 1x / week b. 1x or more / month c. 5 - 10 times d. 2 - 4 times

e. 1 time f. Never

6. In the last year, approximately how many times have you collaborated with (this person) in advocacy or action for health with authorities

a. 1x / week b. 1x or more / month c. 5 - 10 times d. 2 - 4 times

e. 1 time f. Never

**Part 2: Interactions with authorities**

*I am going to ask you some questions about your participation in the interactions with the authorities who were named earlier. I will tell you the name of the authority, and then ask you questions about that person. Then we will go on to the next authority and answer the same questions about him/her, and so on, until we complete the list.*

1. In the last year, approximately how many times have you had contact with (this person) in relation to problems in the health services?

a. 1x / week b. 1x or more / month c. 5 - 10 times d. 2 - 4 times

e. 1 time f. Never

*If Never, skip questions 2 and 3 and go on to the next authority.*

2. How was his/her attitude when facing the problems that you presented?

a. Very open and responsive b. Open c. Neutral d. Negative e. Very negative

f. I don't know

3. How have they responded to the problem(s) presented to them? Have they gotten involved in looking for solutions…

a. Strongly b. Somewhat c. Not at all d. I don't know
